# Circumferential endoscopic submucosal dissection for long-segment Barrettʼs adenocarcinoma: the double-tunnel and single clip-and-loop traction method

**DOI:** 10.1055/a-2058-8202

**Published:** 2023-04-21

**Authors:** Sandro Sferrazza, Federica Crispino, Filippo Vieceli, Andrea Fiorentino, Andrea Michielan, Giovanni de Pretis

**Affiliations:** 1 Gastroenterology and Digestive Endoscopy Unit, Presidio Ospedaliero Santa Chiara, Trento, Italy; 2 Gastroenterology and Hepatology Section, PROMISE, Azienda Ospedaliera Universitaria Policlinico Paolo Giaccone, Palermo, Italy; 3 Gastroenterology and Digestive Endoscopy Unit, Presidio Ospedaliero SantʼOttone Frangipane, Ariano Irpino, Italy

Circumferential endoscopic submucosal dissection (ESD) is an organ-preserving procedure for large areas of esophageal neoplasia. Currently, various endoscopic techniques have been described 1 2 3 4 . Here, we report a successful circumferential ESD of long-segment Barrett’s esophagus (C10M12) using the double-tunnel and single clip-and-loop traction method.

A 67-year-old man with recent demolitive head and neck cancer surgery was referred to our center with anemia. Upper gastrointestinal endoscopy revealed a circumferential adenocarcinoma developing within long-segment Barrett’s esophagus (C10M12). A subsequent computed tomography scan and endoscopic ultrasound were negative for locoregional lymphadenopathy and distal metastases. The treatment options were discussed with the patient at a multidisciplinary meeting, with the option of esophageal ESD being chosen.

ESD was performed with the patient under general anesthesia, using a DualKnife and an insulated tip (IT)-2 DualKnife for mucosal incision and submucosal dissection, respectively ( Video 1 ). After markings had been placed and the submucosa injected, a distal mucosal incision was performed to set up the distal limit of the tunnel. A submucosal tunnel was created on the anterior wall, where good access is obtained owing to the effects of gravity, and was extended in the craniocaudal direction. Subsequently, a posterior tunnel was created with a loop-clip applied on the inferior pillar to facilitate access, which is made more difficult by the effects of gravity, and to expedite submucosal exposure.

**Video 1** Novel placement of an esophageal wound vacuum for persistent anastomotic leak.


A 12-cm circumferential en bloc specimen was removed, with the procedure taking 300 minutes. Histology revealed a squamous cell carcinoma with invasion of the lamina propria (T1) and negative margins (R0). Despite triamcinolone injections into the residual submucosal areas, esophageal stenosis was detected after 12 weeks and treated with two pneumatic dilations up to 15 mm.

To the best of our knowledge, this is the first report of a circumferential ESD performed via the tunneling method with a single clip-band-line traction.

Endoscopy_UCTN_Code_TTT_1AO_2AG

## Notes

Endoscopy E-Videos

https://eref.thieme.de/e-videos
*E-Videos* is an open access online section of the journal *Endoscopy* , reporting on interesting cases and new techniques in gastroenterological endoscopy. All papers include a high-quality video and are published with a Creative Commons CC-BY license. Endoscopy E-Videos qualify for HINARI discounts and waivers and eligibility is automatically checked during the submission process. We grant 100% waivers to articles whose corresponding authors are based in Group A countries and 50% waivers to those who are based in Group B countries as classified by Research4Life (see: https://www.research4life.org/access/eligibility/ ). This section has its own submission website at https://mc.manuscriptcentral.com/e-videos
